# Effect of Cannabidiol (CBD) on Canine Inflammatory Response: An Ex Vivo Study on LPS Stimulated Whole Blood

**DOI:** 10.3390/vetsci8090185

**Published:** 2021-09-04

**Authors:** Enrico Gugliandolo, Patrizia Licata, Alessio Filippo Peritore, Rosalba Siracusa, Ramona D’Amico, Marika Cordaro, Roberta Fusco, Daniela Impellizzeri, Rosanna Di Paola, Salvatore Cuzzocrea, Rosalia Crupi, Claudia Dina Interlandi

**Affiliations:** 1 Department of Veterinary Science, University of Messina, 98166 Messina, Italy; egugliandolo@unime.it (E.G.); plicata@unime.it (P.L.); rcrupi@unime.it (R.C.); claudiadina.interlandi@unime.it (C.D.I.); 2 Department of Chemical, Biological, Pharmaceutical, and Environmental Science, University of Messina, 98166 Messina, Italy; aperitore@unime.it (A.F.P.); rsiracusa@unime.it (R.S.); rdamico@unime.it (R.D.); rfusco@unime.it (R.F.); dimpellizzeri@unime.it (D.I.); 3 Department of Biomedical and Dental Sciences and Morphofunctional Imaging, University of Messina, 98166 Messina, Italy; cordarom@unime.it; 4 Department of Pharmacological and Physiological Science, Saint Louis University School of Medicine, Saint Louis, MO 63104, USA

**Keywords:** cannabidiol, ex vivo, whole dog blood, canine inflammatory response

## Abstract

The use of cannabidiol (CBD) for animal species is an area of growing interest, for example for its anti-inflammatory and immuno-modulating properties, even though all of its biological effects are still not fully understood, especially in veterinary medicine. Therefore, the aim of this study was to investigate the anti-inflammatory and immuno-modulating properties of CBD for the first time directly in canine inflammatory response. We used an ex vivo model of LPS-stimulated whole dog blood. We stimulated the whole blood from healthy dogs with LPS 100 ng/mL for 24 h in the presence or not of CBD 50 and 100 μg/mL. We observed a reduction in IL-6 and TNF-α production from the group treated with CBD, but non-altered IL-10 levels. Moreover, we also observed from the CBD-treated group a reduction in Nf-κB and COX-2 expression. In conclusion, we demonstrated for the first time the anti-inflammatory and immuno-modulating properties of CBD directly in dogs’ immune cells, using a canine ex vivo inflammatory model. The results obtained from these studies encourage further studies to better understand the possible therapeutic role of CBD in veterinary medicine.

## 1. Introduction

Cannabidiol (CBD) is one of the major non-psychoactive plant (*Cannabis* sp.)-derived cannabinoids that has structural similarity to the primary psychotropic congener in cannabis, D9-tetrahydrocannabinol (THC). The use of cannabis for animal species is an area of growing interest, even if to date there are few studies that have characterized the biological effect of CBD in different animal species [1]. CBD is known to act as an anti-inflammatory and immunomodulant agent, and the effects of CBD on immune responses can involve both innate or adaptive responses [2]. Although there are only a few studies on the therapeutic use of CBD in dogs, the pharmacokinetics and tolerability of CBD has been characterized [3,4]. Recently, a pilot study conducted on dogs with naturally occurring osteoarthritis described the safety and efficacy of CBD for symptom relief of canine osteoarthritis-associated pain [5]. However, the immunomodulating, anti-inflammatory and other biological activities of CBD have been characterized much more often in human medicine than in veterinary medicine [6,7,8]. The aim of this study was to evaluate the effect of CBD directly in canine inflammatory response, and to achieve this aim, we used an ex vivo model where whole blood from healthy dogs was stimulated with LPS (100 ng/mL). Ex vivo whole blood stimulation is a reliable method to study the effect of immunomodulatory agents [9,10], as this method replicates the natural environment of immune cell response, preventing changes in phenotype that may occur with the isolation of the cells and minimize the volume of starting blood needed [11]. Thus, this model allowed us to characterize the effect of CBD on canine inflammatory response. As previously seen in other species, CBD is known to modulate cytokines’ secretion from immune cells and thus modulate several pathways in immune function [12,13]. We focused on the levels of the major regulating cytokines, IL-6, TNF-α and IL-10. IL-6 and TNF-α are the major pro-inflammatory cytokines driving inflammatory response and immune cell activation, and IL-10 is an anti-inflammatory cytokine and immune-regulating mediator [14]. Furthermore, we evaluated the effect of CBD on Nf-κB and COX-2 expression. Nf-κB is a key regulator of inflammatory response by regulating the expression of several pro-inflammatory mediators including COX-2. The finding of this study support the anti-inflammatory and immunomodulant effect of CBD in dogs’ immune cells, even though future studies are needed to better understand the biological activity of this compound in different species.

## 2. Materials and Methods

### 2.1. Canine Donors and Blood Collection

Whole blood samples were obtained from 6 healthy dogs (working lines of German Shepherd, aged between 2 and 10 years, 3 male and 3 female). An aliquot of heparinized blood was kindly donated with the owner’s informed consent from dogs undergoing routine blood tests. The dogs were healthy, based on clinical examinations performed by veterinarians and the results of complete blood cell counts. Blood samples were processed within 1 h of collection.

### 2.2. Whole Blood Culture and Stimulation

Whole blood culture and stimulation were performed as previously described [15]. Briefly, blood from each subject was diluted 1:2 with RPMI 1640 (Gibco, Life Technologies Corporation, Waltham, MA, USA) culture medium containing 200 U/mL penicillin and 200 mg/mL streptomycin. An aliquot of 500 μL of diluted whole blood from each subject was incubated (37 °C, 5% CO_2_) for 24 h with or without CBD (kanarescue 5%^©^). CBD, in the form of kanarescue compaun, was dissolved in DMSO (final concentration of 0.1% in assay media for all assays) and added directly to the media at the concentrations indicated as follow in duplicate:

1. Control: whole blood + vehicle (DMSO 0.1%)
2. LPS: whole blood + LPS 100 ng/mL + vehicle (DMSO 0.1%)
3. CBD 50 μg: whole blood + LPS 100 ng/mL + CBD (kanarescue 5% in DMSO 0.1%) 50 μg/mL
4. CBD 100 μg: whole blood + LPS 100 ng/mL CBD (kanarescue 5% in DMSO 0.1%) 100 μg/mL

After incubation, samples were centrifuged at 2000× *g* for 5 min at room temperature and processed as homogenous samples for each condition. In particular, the supernatants were harvested and stored immediately at −20 °C until further use, and cell pellets stored at −20 °C were immediately resuspended in appropriate lysis buffer and processed for rt-PCR and Western blot analysis as described above. 

### 2.3. ELISA Assays

Cytokine evaluation was performed in whole blood culture supernatant using a canine-specific ELISA kit according to the manufacturer’s protocols [16], for IL-6, IL-10 and TNF-α (Quantikine, R&D system). Briefly, 100 μL of the appropriate sample was added into the appropriate well of a 96-well plate and incubated at room temperature for 2 h. After washing, 200 µL of conjugate ab was added to each well and incubated at room temperature for 2 h. After washing, 120 μL of the substrate solution was added for 30′, and then 120 μL of the stop solution was added to each well. Finally, the plates were read at 450 nm. The lower limit detections were, respectively, 31.3, 15.6, and 7.8 pg/mL.

### 2.4. rt-PCR

Total RNA was isolated from cell pellets using the commercially available column kit RNeasy mini kit (Qiagen) according to the manufacturer’s protocols. Briefly, samples were lysed, and subsequently ethanol was added to obtain ideal binding conditions. The lysates obtained were loaded into the RNeasy silica membrane. RNA bound into the column and all contaminants were washed out. Pure, concentrated RNA was eluted in 50 μL of water. RNA was quantified with a spectrophotometer (NanoDrop, NanoReady Life Real, Hangzhou, China). Subsequently, first-strand cDNA synthesis was performed using RevertAid H Minus First Strand cDNA Synthesis Kit (Thermo Scientific™, Rodano (MI), Italy) according to the manufacturer’s protocols and recommendations. The relative abundance of COX-2 was assessed by RT-PCR using QuantiTect SYBR Green PCR Kits (Qiagen, Milano, Italy) and QuantiTect primer Cf_PTGS2_1_SG (Qiagen) on a CFX-96 Touch Deep Well Real-Time PCR Detection System (Bio-Rad, Segrate (MI), Italy) using 1 μL of total cDNA. PCR conditions were as follows: initial denaturation at 95 °C for 15 min, followed by 45 cycles of amplification at 94 °C for 15 s and 72 °C for 30 s. Final extension at 60 °C for 60 s and holding at 4 °C were then performed. To provide precise quantification of the initial target in each PCR reaction, the amplification plot was examined, and the point of the early log phase of product accumulation was defined by assigning a fluorescence threshold above the background, defined as the threshold cycle number or Cq. Differences in the threshold cycle number were used to quantify the relative amount of the PCR targets contained within each tube with CFX Maestro Software (Bio-Rad). The relative expression of different amplicons was calculated by the delta-delta Cq (ΔΔCq) method and converted to the relative expression ratio (2−ΔΔCq) for statistical analysis. All canine data were normalized to the endogenous reference genes β-actin and GAPDH combined, respectively, Cf_ACTB1_1_SG and Cf_GAPDH_1_SG (QuantiTect primers, Qiagen). For each target gene, besides the biological replicates, three technical replicates were performed. Negative controls using RNA as a template were also included in all runs to test for the possible genomic DNA contamination of the samples.

### 2.5. Western Blot

Western blot analysis was performed as previously described in our previous studies with minor changes [17,18,19]. Briefly, protein concentrations were determined by the Bio-Rad protein assay method using bovine serum albumin as standard. Samples were heated at 100 °C for 5 min, and equal amounts of protein were separated on SDS-PAGE gel and transferred to PVDF membranes. The membranes were blocked with 1 × phosphate-buffered saline (PBS) and 5% (*w/v*) non- fat dried milk for 40 min at room temperature, and then blots were incubated with specific primary antibody Anti-Nf-κB p65 (1:1000, Cell Signalling) or anti-beta actin (1:1000, Abcam) in 1 × PBS, 5% *w/v* non-fat dried milk, and 0.1% Tween-20, and incubated at 4 °C overnight. After that, blots were incubated with the peroxidase-conjugated bovine anti-mouse IgG secondary antibody or peroxidase-conjugated goat anti-rabbit IgG (1:2000, Jackson Immuno Research) for 1 h at room temperature. The relative expression of the protein bands was detected with an enhanced chemiluminescence (ECL) system (Bio-Rad) and visualized with the Chemi Doc XRS (Bio-Rad, Segrate (MI), Italy). The bands were analyzed by using Image Lab 3.0 software (Bio-Rad, Segrate (MI), Italy) and standardized to the relevant housekeeping protein level. Results are expressed with the relative fold change to the control group.

### 2.6. Statistical Analysis

Each experiment included three independent replicates. The data resulting from all experiments are expressed as means ± SEM. Statistical differences between groups were compared using one-way ANOVA, followed by Tukey’s post hoc test for multiple comparisons, using GraphPad Prism version 9 (GraphPad Software Inc., La Jolla, CA, USA). A *p*-value of less than 0.05 was considered statistically significant [20,21].

## 3. Results

### 3.1. Effect of CBD on IL-6 and TNF-α Response in LPS Stimulated Whole Blood

**Figure 1:**
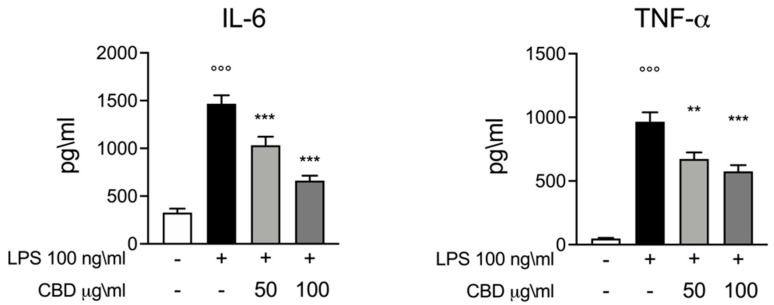
ELISA assay for IL-6 and TNF-α whole blood culture supernatant 24 h after LPS 100 ng/mL stimulation. Data are presented as means ± SEM for each group. °°° *p* < 0.01 vs. control; ** *p* < 0.01 vs. LPS; *** *p* < 0.001 vs. LPS.

Consistent with previous publications, we used an ex vivo model of inflammation where whole blood was stimulated with a canonical inflammatory stimulus such as LPS (100 ng/mL for 24 h) to induce an inflammatory response. Cytokines are the driving factors for inflammatory and immune response. Thus, we decided to evaluate the secretion of two of the major pro-inflammatory mediators, IL-6 and TNF-α, in the different experimental conditions used in this study. The ELISA test performed in the whole blood culture supernatant for IL-6 and TNF-α showed a significant increase in the secretion of these pro-inflammatory cytokines compared with the LPS-stimulated group, serving as our positive control group. As shown in Figure 1, when stimulation with LPS was performed in the presence of CBD (50 and 100 μg/mL) we observed a significant reduction in cytokines secretion. We observed a dose dependence effect of CBD; when compared to CBD 50 μg/mL, the dose of CBD 100 μg/mL produced a bigger inhibitory effect on IL-6 and TNF-α release in whole blood supernatant.

### 3.2. Effect of CBD on IL-10 in LPS Stimulated Whole Blood

**Figure 2:**
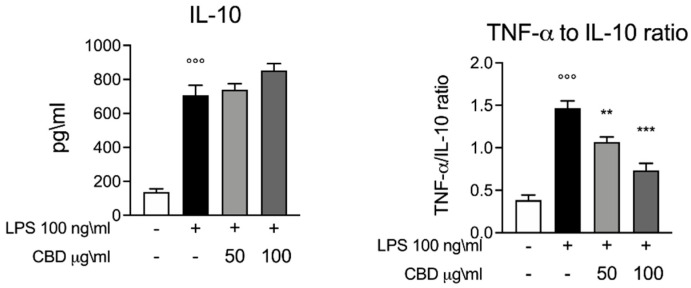
ELISA assay for IL-10. Left panel, whole blood culture supernatant 24 h after LPS 100 ng/mL stimulation. Right panel, TNF-α to IL-10 ratio graph calculated based on the results of the ELISA assay. Data are presented as means ± SEM for each group. °°° *p* < 0.01 vs. control; ** *p* < 0.01 vs. LPS; *** *p* < 0.001 vs. LPS.

Next, we evaluated the trend of IL-10 as a major anti-inflammatory cytokine. Furthermore, IL-10 plays a key role in the function regulation of several immune cells. Consistent with previous studies, the whole blood stimulation with LPS induced an increase in the levels of IL-10 compared to the control [15,22]. Interestingly, we found that CBD did not alter the levels of IL-10 (Figure 2); instead, we observed a slight trend in IL-10 levels after the treatment with CBD in a dose-dependent manner. As shown in Figure 2, we evaluated the TNF-α to IL-10 ratio to highlight a shift in inflammatory phenotype [22]. Our results show a significant increase in the TNF-α to IL-10 ratio induced by LPS, while the CBD produces a significant decrease in the TNF-α to IL-10 ratio in a dose-dependent manner. 

### 3.3. Effect of CBD on Nf-κB and COX-2 Induction in LPS Stimulated Whole Blood

**Figure 3:**
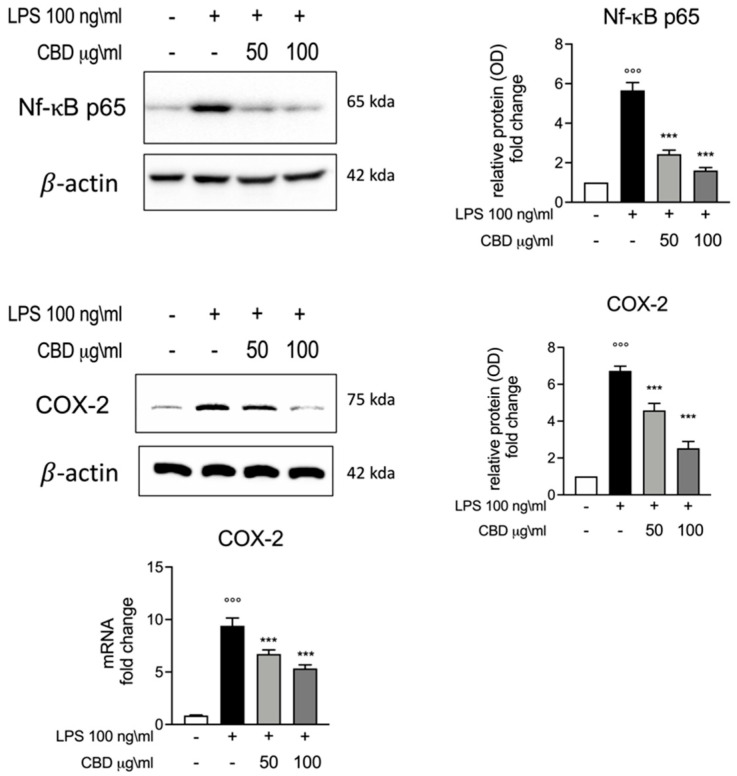
Upper panels, Western blot analysis for Nf-κB expression and relative comparative fold change graph. Lower panel, Western blot and rt-PCR analysis for COX-2 protein and mRNA expression among the different experimental groups. Data are presented as means ± SEM for each group. °°° *p* < 0.01 vs. control; *** *p* < 0.001 vs. LPS.

We also evaluated the effect of CBD on inflammatory pathways through the evaluation of Nf-κB and COX-2 expression levels. NF-κB activation has significant effects on the regulation of inflammatory response and in the transcriptional regulation of many pro-inflammatory cytokines, including TNF-α and IL-6. As is widely known, the LPS stimuli induced an increase in Nf-κB p-65 activation. Through Western blot analysis, we highlighted the expression of Nf-κB p-65; as shown in Figure 3, we observed a significantly increase in Nf-κB p-65 expression in the LPS group. According to the results for IL-6 and TNF-α (Figure 1), we observed a reduction in Nf-κB expression for the groups incubated with LPS and CBD (50 and 100 μg/mL) compared to the LPS-only group. Subsequently we evaluated the mRNA and protein levels for COX-2, which is known to be upregulated during inflammation. COX-2 plays a fundamental role in the inflammatory cascade, and thus in acute and chronic inflammation. Additionally, for COX-2, we observed significantly increased levels of both mRNA and protein for the LPS group, while the treatment with CBD (50 and 100 μg/mL) showed significantly lower COX-2 mRNA and protein expression compared to LPS. These results are in accordance with the results observed for Nf-κB and confirm an immune regulatory effect for CBD.

## 4. Discussion

CBD is the major non-psychoactive component of *Cannabis* sp.; even though many of its biological effects are not clearly understood, recently, CBD was authorized in some countries for therapeutic purposes for several clinical conditions [3]. To date, only a few studies have assessed the therapeutic efficacy of CBD in dogs. CBD has been seen to be efficacious in reducing pain and joint inflammation signs in dogs with osteoarthritis [23], and in dogs with epilepsy [24]. As synthetized in a review by Della Rocca [3], despite the limited data on the pharmacological effects of CBD in dogs, the pharmacokinetics and tolerability of CBD and those of cannabis derivatives in general have been evaluated. Evidence supports the immunomodulant proprieties of CBD [25,26,27,28], but to date, there are no scientific data on the direct immunomodulatory effect of CBD in dogs. In fact, the biological activities of CBD have been characterized much more often in humans, with more clinical trials, than in other animal species [29,30,31]. Thus, the aim of this study was to assess the immunomodulatory and anti-inflammatory effect of CBD directly in dogs. Thus, we used an ex vivo model of whole dog blood from healthy dogs stimulated with LPS. This is a reliable model to study the effect of immune modulatory agents or immune response [32] in target species, and it has previously been demonstrated that this model is more representative of in vivo cytokine response patterns [33]. LPS as canonical inflammatory stimulus which is able to evoke a pro-inflammatory phenotype of immune cells [34]. Cytokines are responsible for the regulation of immunological and inflammatory processes, and play an important role in the proliferation and differentiation of lymphoid cells. Our experimental protocols consist of the stimulation of healthy dogs’ whole blood with LPS 100 ng/mL in the presence or not of CBD 50 and 100 μg/mL. We started by evaluating the levels of the pro-inflammatory cytokines IL-6 and TNF-α and of the anti-inflammatory IL-10 in the whole blood culture supernatant through ELISA assays. IL-6 and TNF-α play a key role in the physiological inflammatory response such as during infection, but also in several diseases where elevated levels of these cytokines are associated with a poor clinical outcome. IL-6 regulates both the innate and adaptive immune response [35], is a well-consolidated marker of inflammation, such as systemic inflammation, sepsis, and autoimmune disease [36], and is also a prognostic marker for canine critical care patients with acute internal disease [37]. Consistent with previous studies, we found that LPS stimulation of canine whole blood induces a significant increase in the secretion of IL-6 and TNF-α [15]. Our results show that whole dog blood stimulated with LPS and treated with CBD 50 and 100 μg/mL had a significant lower level of IL-6 and TNF-α compared to LPS. These findings are in accordance with previous studies that assessed the immune modulatory effect of CBD in humans [28,38], due to the proprieties of CBD. To better understand the effect of CBD on dog immune cells, we decided to investigate the effect not only on the pro-inflammatory cytokines IL-6 and TNF-α, but also on IL-10 as an anti-inflammatory and immunoregulating cytokine. Interestingly, our results show that differently from IL-6 and TNF-α, CBD did not alter the levels of IL-10 in response to LPS; this effect of CBD has also been observed in previous studies [39,40]. In particular, we observed a slight trend for the upregulation of IL-10 by CBD—this can partially be explained by the combination of CBD treatment and LPS stimulation [41]. The balance between TNF-α and IL-10 is important for immune homeostasis maintenance; commonly, an increase in TNF-α is counterbalanced by a simultaneous increase in the anti-inflammatory cytokine IL-10, which suppresses the production of many activating and regulatory mediators [42,43]. Thus, we also evaluated the TNF-α to IL-10 ratio as a cytokine marker of the balance between key pro- and anti-inflammatory levels [12,18,40]. Our results show that LPS stimulation produces a significant increase in the TNF-α to IL-10 ratio in whole dog blood, while treatment with CBD is able to restore the TNF-α to IL-10 ratio compared to the control groups. To better investigate the effect of CBD during inflammation, we evaluate the expression levels of Nf-κB p-65. NF-κB regulates several events in the innate and adaptive immune response and serves as a key regulator of inflammatory responses. 

NF-κB induces the expression of several pro-inflammatory genes, including those encoding cytokines and chemokines [44]. Our results show that treatment with CBD 50 and 100 μg/ mL reduced Nf-κB p65 expression during stimulation with LPS. COX-2 is a key enzyme in the synthesis of prostaglandins from arachidonic acid and plays a key role in inflammatory response. Its expressions is known to be upregulated in response to certain stimuli, including growth factors and pro-inflammatory cytokines [45]. Our results show that stimulation of LPS in healthy whole dog blood induced a significant increase in COX-2 mRNA, while the treatment with CBD 50 and 100 μg/mL reduced the increased COX-2 mRNA induced by LPS. This effect on COX-2 can be explained by the results observed for IL-6, TNF-α and Nf-κB.

## 5. Limitations of the Study 

Due to the novelty of this preliminary study, there are some limitations which should be pointed out. Although some limited studies published previously show that CBD is well-tolerated in different species in vivo, the lack of more accurate studies on the cytotoxic effects of this compound, if present, and in particular on the effects on different cell types means that there is no indication as to whether the doses used in this study could fall within a therapeutic range. A limitation of this study is the limited range of CBD concentrations used, and the limited sample size in terms of the dog breeds used. Furthermore, with regard to the sources and preparation of CBD, they are very different from each other, both in terms of composition and the concentration of CBD, which is often not well titrated. In this study, we refer to CBD as originating from a commercial preparation available for dogs, such as kanarescue 5%.

## 6. Conclusions

In conclusion, in this study, we provide the first evidence of the immunomodulatory effect of CBD in stimulated whole dog blood. Our results demonstrate that CBD was able to reduce the inflammatory response induced by LPS. In particular, we found that CBD was able to modulate the cytokine production stimulated by LPS and thus reduced the inflammatory response, as confirmed by Nf-κB and COX-2 reduction in the group treated with CBD 50 and 100 μg/mL. Due to the lack of scientific data on the use of CBD in dogs, our results obtained in an ex vivo canine model encourage further studies to better understand the possible therapeutic role of CBD in veterinary medicine.

## Data Availability

All data obtained from this study are included into the manuscript.
